# Limited Anti-Inflammatory Role for Interleukin-1 Receptor Like 1 (ST2) in the Host Response to Murine Postinfluenza Pneumococcal Pneumonia

**DOI:** 10.1371/journal.pone.0058191

**Published:** 2013-03-06

**Authors:** Dana C. Blok, Koenraad F. van der Sluijs, Sandrine Florquin, Onno J. de Boer, Cornelis van ’t Veer, Alex F. de Vos, Tom van der Poll

**Affiliations:** 1 Center of Experimental and Molecular Medicine, Center of Infection and Immunity Amsterdam, Academic Medical Center, University of Amsterdam, Amsterdam, The Netherlands; 2 Laboratory of Experimental Intensive Care and Anesthesiology, Academic Medical Center, University of Amsterdam, Amsterdam, The Netherlands; 3 Department of Pathology, Academic Medical Center, University of Amsterdam, Amsterdam, The Netherlands; 4 Division of Infectious Diseases Academic Medical Center, University of Amsterdam, Amsterdam, The Netherlands; Hannover School of Medicine, Germany

## Abstract

Interleukin-1 receptor like 1 (ST2) is a negative regulator of Toll-like receptor (TLR) signaling. TLRs are important for host defense during respiratory tract infections by both influenza and *Streptococcus (S.) pneumoniae*. Enhanced susceptibility to pneumococcal pneumonia is an important complication following influenza virus infection. We here sought to determine the role of ST2 in primary influenza A infection and secondary pneumococcal pneumonia. ST2 knockout (*st2* ^−/−^) and wild-type (WT) mice were intranasally infected with influenza A virus; in some experiments mice were infected 2 weeks later with *S. pneumoniae*. Both mouse strains cleared the virus similarly during the first 14 days of influenza infection and had recovered their weights equally at day 14. Overall *st2^−/−^* mice tended to have a stronger pulmonary inflammatory response upon infection with influenza; especially 14 days after infection modest but statistically significant elevations were seen in lung IL-6, IL-1β, KC, IL-10, and IL-33 concentrations and myeloperoxidase levels, indicative of enhanced neutrophil activity. Interestingly, bacterial lung loads were higher in *st2^−/−^* mice during the later stages of secondary pneumococcal pneumonia, which was associated with relatively increased lung IFN-γ levels. ST2 deficiency did not impact on gross lung pathology in either influenza or secondary *S. pneumoniae* pneumonia. These data show that ST2 plays a limited anti-inflammatory role during both primary influenza and postinfluenza pneumococcal pneumonia.

## Introduction

Influenza virus is a common respiratory pathogen affecting all ages [1]. On average 10–15% of the United States population is infected by influenza annually. Although most subjects infected with influenza recover uneventfully, each year seasonal influenza epidemics are responsible for more than 200.000 hospitalizations and associated with more than 30.000 deaths in the United States [2]. Influenza related severe illness and death of otherwise healthy individuals are often attributable to bacterial superinfection of the lung [3]. In addition to being the most prevalent cause of community-acquired pneumonia, *Streptococcus (S.) pneumoniae* is also most frequently reported as the causative agent involved in secondary bacterial pneumonia following influenza infection [4]–[6].

Insight into the underlying mechanisms contributing to the enhanced susceptibility to bacterial pneumonia due to prior influenza infection is gradually growing, implicating viral, bacterial and host factors [4], [7]–[10]. Several studies have linked Toll-like receptors (TLRs) to host defense during respiratory tract infection by influenza and/or *S. pneumoniae*. TLRs are pattern recognition receptors that play a key role in innate immunity by virtue of their capacity to recognize conserved motifs expressed by pathogens [11]. Influenza virus RNA is recognized by TLR3 and TLR7, resulting in the induction of a protective type-I interferon (IFN) response [12]–[14]. In accordance, mice deficient for TLR3 and TLR7 (*tlr3/tlr7* ^−/−^) or the common TLR adapter MyD88 (*myd88* ^−/−^) demonstrated an increased lethality upon infection with influenza [15]. Cell wall components of *S. pneumoniae* are primarily recognized by TLR2 [16],[17], while TLR4 is the receptor for pneumolysin, an important virulence factor expressed by the pneumococcus [18], [19]. TLR2 and TLR4 may act together with TLR9, the receptor that recognizes bacterial DNA, in inducing cytokine release by inflammatory cells upon exposure to *S. pneumoniae* [20]. In vivo, especially TLR9 was reported to be important for host defense during pneumococcal pneumonia, as reflected by increased bacterial growth and lethality in *tlr9* ^−/−^ mice [21]; *myd88* ^−/−^ mice displayed a similar hypersusceptible phenotype [22]. Importantly, influenza induced a sustained desensitization of murine alveolar macrophages to TLR ligands resulting in reduced inflammation and cell recruitment when confronted with a secondary bacterial lung challenge [23]. Similarly, human peripheral blood mononuclear cells isolated from H1N1 influenza infected patients failed to generate adequate TNF-α and IFN-γ levels upon exposure to *S. pneumoniae,* signifying a vulnerability of influenza infected persons to pneumococcal superinfection [24]. On the other hand, excessive TLR activation may contribute to the enhanced lung pathology accompanying fatal postinfluenza pneumococcal pneumonia [25]. Together these data point to an important role of TLR signaling in the host response to influenza, *S. pneumoniae* and postinfluenza pneumococcal pneumonia.

Uncontrolled stimulation of TLRs can lead to disproportionate inflammation and tissue injury. Several negative regulators of TLRs designated to prevent excessive TLR activation have been identified, including Interleukin-1 receptor like 1 (IL1RL1 also known as ST2) [26], [27]. ST2 inhibits MyD88 dependent signaling, thereby dampening the immune response to multiple TLR ligands [26]. Arguably, ST2 could impact on the host response to postinfluenza pneumococcal pneumonia in two ways: ST2 could be involved in the reduced responsiveness of immune cells to TLR ligands upon exposure to influenza [23] making the host more vulnerable to a secondary hit and/or ST2 could oppose the exacerbated lung inflammation during pneumococcal pneumonia following influenza [25] thus preventing damage and promoting lung integrity. We here sought to determine the role of ST2 in respiratory tract infection by *S. pneumoniae* following influenza using *st2* ^−/−^ mice and our established model of postinfluenza pneumonia [28]–[31].

## Materials and Methods

### Animals

Specific pathogen free 9–11 week old BALB/c mice (wild-type [WT]) were purchased from Charles River (Maastricht, The Netherlands). *St2* ^−/−^ mice [32] backcrossed eight times to a BALB/c background were kindly provided by dr. Andrew N.J. McKenzie (MRC Laboratory of Molecular Biology, Cambridge, United Kingdom) and bred in the animal facility of the Academic Medical Center in Amsterdam. Age- and sex-matched animals were used in all experiments. The Animal Care and Use Committee of the University of Amsterdam approved all experiments.

### Experimental Infections

The model of postinfluenza pneumococcal pneumonia has previously been described in detail [28]–[31]. In short, influenza A/PR/8/34 (ATCC VR-95, Rockville, MD) was grown in LLC-MK2 cells. Mice were anesthetized by inhalation of isoflurane (Abbott Laboratories, Kent, UK) and inoculated intranasally with 50 µl phosphate buffered saline containing 10xTCID50 (50% tissue culture infective dose). After 3, 8 or 14 days mice were euthanized and viral loads determined in lung homogenates using real-time quantitative polymerase chain reaction (PCR) [33]. Pneumococcal pneumonia was induced 14 days post inoculation with Influenza A by intranasal inoculation with 50 µl normal saline containing 1.5×10^4^ colony-forming units (cfu) of *S. pneumoniae* (serotype 3; American Type Culture Collection, ATCC 6303, Rockville, MD). Mice were sacrificed 6, 24 or 48 hours after inoculation with *S. pneumoniae*; blood, lung, spleen and liver were harvested for quantitative bacterial cultures exactly as described [28]–[31].

### Histopathological Analysis

Lungs were fixed in 10% formalin and embedded in paraffin. Four micrometer lung sections were stained with hemotoxylin and eosin (H&E) and analyzed by a pathologist who was blinded to the groups. To score lung inflammation and damage, a semiquantitative scoring system was used. For this the entire lung surface was analyzed with respect to the following parameters: pleuritis, bronchitis, edema, interstitial inflammation, percentage of pneumonia, and endothelialitis. Each parameter was graded on a scale of 0 to 4 with 0 as ‘absent’ and 4 as ‘severe’. The total “lung inflammation score” was expressed as the sum of the scores for each parameter, the maximum being 24. Granulocyte staining with Ly-6G monoclonal antibody (BD Pharmingen, San Diego, CA, USA) of four micrometer lung sections was done as described previously [34], [35]. The entire Ly-6G stained lung sections were digitized with a slide scanner (Olympus, Tokyo, Japan) using the 10× objective and subsequently exported as TIFF images. Immunupositive areas were analyzed with ImageJ (version 2006.02.01, US National Institutes of Health, Bethesda, MD) and expressed as the percentage of the total lung surface area [36].

### Assays

Lung homogenates for cytokine and other measurements were prepared as described [28]–[31]. Myeloperoxidase (MPO), tumor necrosis factor (TNF)-α, interleukin (IL)-1β, IL-6, IL-10, IL-33, IL-5, IL-13, macrophage inflammatory protein (MIP)-2, cytokine-induced neutrophil chemo attractant (KC), IFN-γ and amphiregulin were measured using specific enzyme-linked immunosorbent assays (MPO: Hycult, Uden, the Netherlands; all other: R&D systems, Abingdon, UK) in accordance with the manufacturer’s recommendations.

### Statistical Analysis

Data are expressed as box and whisker diagrams depicting the smallest observation, lower quartile, median, upper quartile and largest observation or as bar graphs depicting means ± SEM. Differences were analyzed by Mann Whitney U test or Chi square test. A value of *P*<0.05 was considered statistically significant.

## Results

### Body Weight and Viral Clearance during Primary Influenza Infection

**Figure 1:**
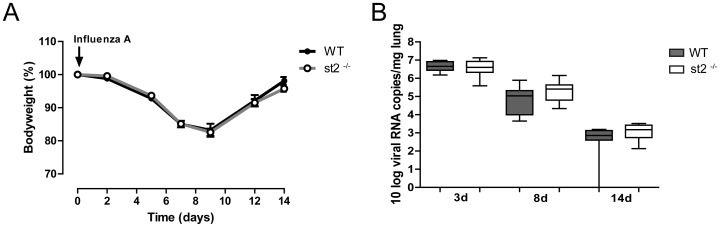
Body weight and viral load of WT and *st2* ^−/−^ mice during influenza pneumonia. WT *(solid circles/whiskers)* and *st2* ^−/−^ mice *(open circles/whiskers)* were infected intranasally with influenza A at t = 0. (A) Body weight changes relative to weight (100%) prior to inoculation. Data are mean ± SEM of 8 mice per group; weight curve displays measurements of 4 separate experiments. (B) Viral RNA copies per mg lung. Data are expressed as box-and-whisker diagrams depicting the smallest observation, lower quartile, median, upper quartile and largest observation of eight mice per group.

The primary objective of our study was to determine the role of ST2 in secondary pneumococcal pneumonia following influenza A infection. In order to adequately address this study question, we first determined the contribution of ST2 to the host response during primary influenza A. *St2* ^−/−^ and WT mice were infected with influenza A intranasally and body weights (Figure 1A) and viral loads (Figure 1B) were determined at indicated time points during the subsequent 14 days. As reported earlier [29], [37], [38], influenza caused a transient weight loss, which was most pronounced after 8 days; body weights had recovered at day 14. The temporary changes in body weight were similar in *st2* ^−/−^ and WT mice. Likewise, viral loads, measured 3, 8 and 14 days after infection, were similar in both mouse strains. The uniformity of both weight loss and viral clearance negated the possibility of large baseline differences (*i.e.* prior to infection with *S. pneumoniae*) between *st*2^−/−^ and WT mice.

### Lung Inflammation during Primary Influenza Infection

**Figure 2:**
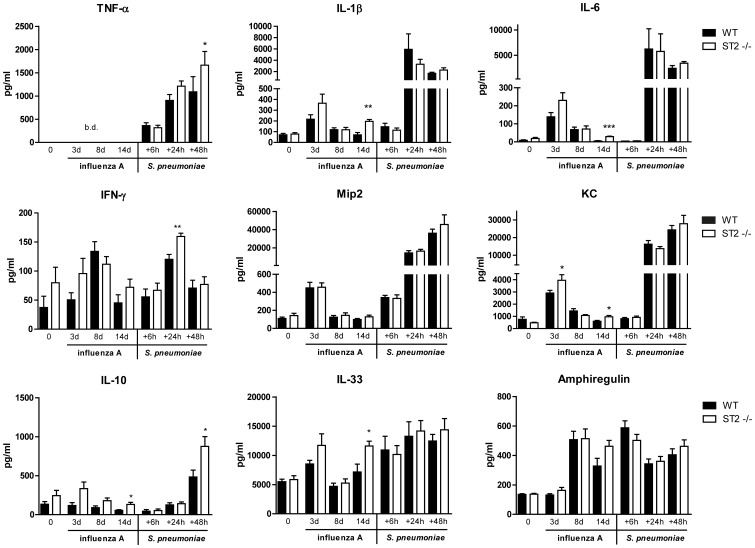
Cytokine and chemokine concentrations in lung of WT and *st2* ^−/−^ mice during (post)influenza pneumonia. Pulmonary levels of TNF-α, IL-1β, IL-6, IFN-γ, MIP-2, KC, IL-10, IL-33 and amphiregulin from WT (solid bars) and *st2* ^−/−^ (open bars) lung homogenates during (post)influenza pneumonia. Data are mean ± SEM of seven to eight mice per group at each time point. *P<0.05, **P<0.01, ***P<0.001 versus WT. b.d., below detection level. TNF-α lower limit of detection: 7 pg/ml.

To determine the possible impact of ST2 on pulmonary inflammation during influenza infection, we measured lung levels of cytokines (IFN-γ, TNF-α, IL-1β, IL-6, IL-10, IL-33, IL-13, IL-5) and chemokines (KC, MIP-2) at 3, 8 and 14 days following influenza inoculation (Figure 2). With the exception of IFN-γ, lung levels of all these cytokines and chemokines were relatively low compared to levels induced by secondary pneumococcal pneumonia (see below, for comparison also shown in Figure 2). Overall, *st2* ^−/−^ mice tended to have higher mediator levels in lungs than WT mice; especially 14 days after infection with influenza A modest but statistically significant differences were seen in pulmonary IL-6 (*P*<0.001), IL-1β (*P*<0.01), KC (*P*<0.05), IL-10 (*P*<0.05) and IL-33 (*P*<0.05) concentrations. TNFα remained below the limit of detection during influenza infection, whereas IL-5 and IL-13 were not induced by influenza infection (data not shown). IL-5 and IL-13 can be produced by recently identified ST2 positive lineage negative lymphoid cells designated type 2 innate lymphoid cells (ILC2) [39]–[41]. These cells also secrete amphiregulin upon ST2 ligation by IL-33. Lung amphiregulin levels increased upon influenza infection but did not differ between *st2* ^−/−^ and WT mice (Figure 2).

**Figure 3:**
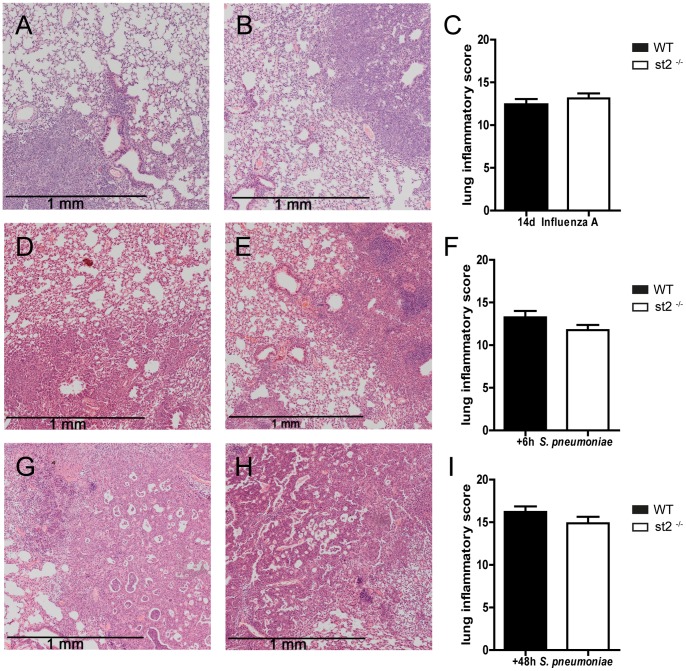
Histopathology of lungs from WT and *st2* ^−/−^ mice during (post)influenza pneumonia. Haematoxylin-eosin staining and semiquantitative histology scores of lung slides, as determined by the scoring system described (Materials and Methods), from WT (solid bars) and *st2* ^−/−^ mice (open bars) 14 days following influenza A infection (A-C) and 6 (D-F) & 48 hours (G-I) post secondary pneumococcal infection. Representative lung slides of WT (A,D,G) and *st2* ^−/−^ mice (B,E,H;). Histology scores are mean ± SEM of 7 to 8 mice per group at each time point.

**Figure 4:**
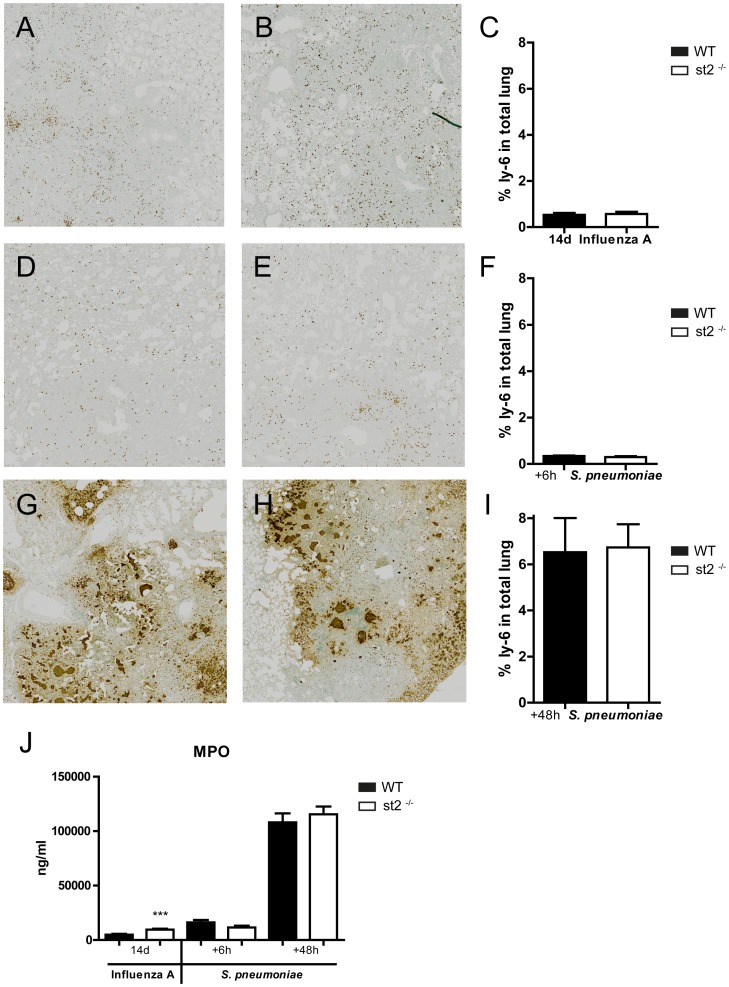
Neutrophil influx into lungs of WT and *st2* ^−/−^ mice during (post)influenza pneumonia. Neutrophil numbers in lung tissue were evaluated by (A–I) Ly-6 staining of lung slides and (J) measurement of MPO levels in lung homogenates obtained from WT (solid bars) and *st2* ^−/−^ mice (open bars) 14 days after influenza A infection (A–C) and 6 (D–F) & 48 hours (G–I) after secondary *S. pneumoniae* infection. The depicted Ly-6 stained lung sections are original magnification ×10 and are representative for 7–8 mice per group. Ly-6 scores (C, F, I) and lung MPO levels are expressed as mean ± SEM of 8 mice per group. ***P<0.001 as compared to WT mice.

To obtain further insight in the role of ST2 in the regulation of lung inflammation induced by influenza A, we semi-quantitatively analyzed lung tissue slides prepared 14 days after viral infection (*i.e.* directly prior to induction of bacterial pneumonia), using the scoring system outlined in the Methods section. No difference in lung inflammation scores was observed between *st2* ^−/−^ and WT mice (Figure 3A–C). Neutrophil presence in the lung prior to secondary bacterial infection was evaluated by counting the number of Ly-6+ cells in lung tissue slides 14 days after viral infection (Figure 4A–C) and by measuring MPO concentrations in the corresponding whole lung homogenates (Figure 4J). Pulmonary MPO levels were slightly but statistically significantly higher in *st2* ^−/−^ mice (*P*<0.001 versus WT mice). However, Ly-6 staining of lung slides did not reveal a similar difference (Figure 4).

### Bacterial Loads and Viral Titers during Postinfluenza Pneumonia

**Figure 5:**
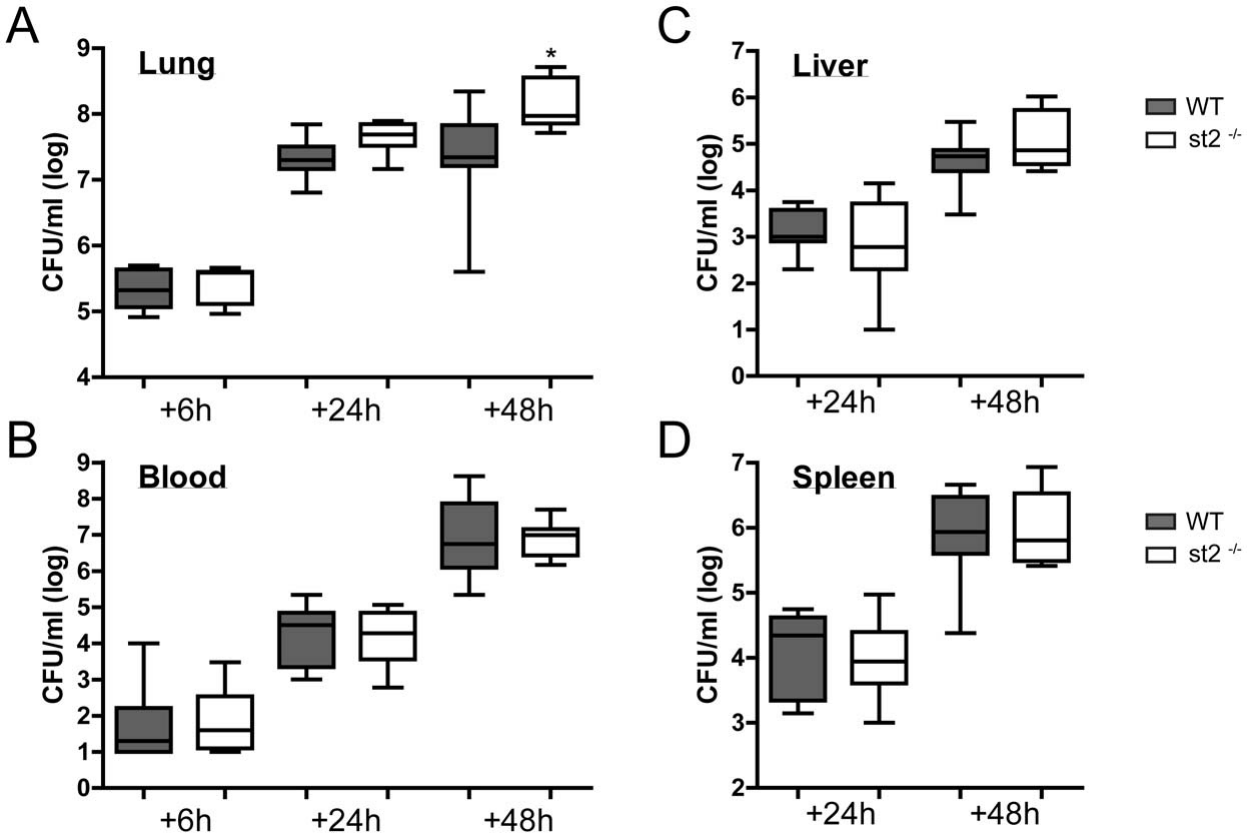
Bacterial loads in lungs of WT and *st2* ^−/−^ mice during postinfluenza pneumonia. WT (solid whiskers) and *st2* ^−/−^ mice (open whiskers) were infected with 1.5×10^4^ of *S. pneumoniae* 14 days post influenza A inoculation. Mice were sacrificed at 6, 24 or 48 hours after secondary infection and bacterial loads were determined in Lung (A), Blood (B), Liver (C) and Spleen (D). Data are box-and-whisker diagrams depicting the smallest observation, lower quartile, median, upper quartile and largest observation. N = 8 per goup at each time point. *P<0.05 versus WT mice.

14 days after infection with influenza A, *st2* ^−/−^ and WT mice were intranasally infected with *S. pneumoniae* and euthanized 6, 24 or 48 hours later to obtain insight into the host immune response. Weight loss, as an indicator of disease severity, was extensive during secondary bacterial pneumonia but did not differ between groups (48 hours after bacterial infection: *st2* ^−/−^ mice 81.3±1.2% of body weight prior to infection with influenza A; WT mice 82.1±2.6%). To determine the role of ST2 in the growth and dissemination of *S. pneumoniae* in the setting of postinfluenza pneumonia, we measured bacterial loads in lung, blood and distant organs (liver and spleen) at the time points indicated (Figure 5). When compared with WT mice, *st2* ^−/−^ mice displayed higher bacterial counts in their lungs at 24 (*P* = 0.055) and 48 hours (*P*<0.05) after infection with *S. pneumoniae*. Bacterial burdens did not differ between groups at distant body sites. Remarkably, *st2* ^−/−^ mice demonstrated an accelerated clearance of influenza A during secondary bacterial infection. Indeed, at 6 hours after infection with *S. pneumoniae*, pulmonary viral loads were lower in *st2* ^−/−^ mice (994±445 copies/mg) than in WT mice (1965±961 copies/mg, *P*<0.05); at later time points more *st2* ^−/−^ than WT mice had undetectable viral loads in their lungs, significantly so at 48 hours after bacterial infection (7/8 versus 2/7 respectively, *P*<0.05 by Chi square test).

### Lung Inflammation during Post Influenza Pneumonia

To obtain insight into the role of ST2 in the regulation of lung inflammation during postinfluenza pneumococcal pneumonia, we measured amphiregulin, cytokines (IFN-γ, TNFα, IL-1β, IL-6, IL-10, IL-33, IL-5, IL-13) and chemokines (KC, MIP-2) in lungs of mice 6, 24 and 48 hours after secondary bacterial infection (Figure 2). Overall, mediator levels did not differ between *st2* ^−/−^ and WT mice, although *st2* ^−/−^ mice had modestly higher levels of IFN-γ at 24 hours (*P*<0.01) and TNF-α (*P*<0.05) and IL-10 at 48 hours (*P*<0.05). Next, we analyzed lung tissue slides obtained 6 or 48 hours after infection with *S. pneumoniae* using the semi-quantitative scoring system described in the Methods section. No differences in total inflammatory scores between WT and *st2* ^−/−^ mice were observed (Figure 3D–I). To evaluate neutrophil influx to the lung during postinfluenza pneumococcal pneumonia we determined MPO levels in lung homogenates and the number of Ly6+ cells in lung tissue slides; no differences between *st2* ^−/−^ and WT mice were found (Figure 4D–J).

## Discussion

It is well appreciated that bacterial superinfections of the lung are a common cause of influenza related complications and death [7]–[9]. Knowledge regarding interactions between the influenza virus, *S. pneumoniae* and host immunity has steadily increased over the last decades. Evidence suggests that a preceding viral respiratory tract infection influences host immunity to such an extent that it is unable to react adequately to a subsequent bacterial challenge, leading to a higher bacterial burden, more invasive disease and a more aggressive inflammatory response than a primary infection with the same organism would evoke [4], [5], [10], [42], [43]. As primary sensors and initiators of innate immunity, TLRs have been implicated in host defense mechanisms during influenza infection [12]–[15], as well as pneumococcal pneumonia in the previously healthy [21], [22] and influenza infected host [25]. We here investigated the potential role of the negative TLR regulator ST2 in host defense against influenza and postinfluenza pneumococcal pneumonia. ST2 was found to exert anti-inflammatory effects during both influenza infection and *S. pneumoniae* pneumonia following influenza infection, which was accompanied by a modestly attenuated growth of pneumococci in ST2 sufficient mice previously exposed to influenza when compared with animals lacking this receptor.

ST2 is a member of the IL-1 receptor family that exists in two forms: a transmembrane full-length form (ST2L) and a soluble, secreted form (soluble ST2) [44]. ST2 is expressed by many hematopoietic cells, Th2 lymphocytes, natural killer (NK) and NKT cells, mast cells, monocytes, macrophages, dendritic cells and granulocytes. Together with IL-1 receptor accessory protein ST2 forms the receptor for IL-33 [45]. Furthermore, it serves an important negative regulatory function in TLR signaling, as reflected by enhanced cytokine release by *st2* ^−/−^ macrophages upon stimulation with TLR agonists [26]. Prior infection with influenza may influence TLR signaling in response to *S. pneumoniae* in different ways. For instance, mice infected with influenza 4–6 weeks earlier displayed a markedly reduced airway responsiveness to various TLR ligands as reflected by an attenuated neutrophil recruitment into the pulmonary compartment [23]. Alveolar macrophages were shown to be responsible for the long-lived reduced responsiveness in lungs previously exposed to influenza. This diminished sensitivity to TLR stimulation resulted in an impaired innate immune response to *S. pneumoniae* accompanied by an enhanced bacterial growth and dissemination [23]. On the other hand, uncontrolled TLR stimulation may contribute to the disproportionate inflammation found in the lungs during postinfluenza pneumococcal pneumonia. Indeed, although TLR2 was not involved in the exaggerated lung inflammation elicited by *S. pneumoniae* in mice pre-exposed to influenza [25], [29], the pulmonary immunopathology and lethality provoked by cell wall components of pneumococci were found to be mediated, at least in part, by this receptor [25]. Hence, ST2, as an inhibitor of MyD88 dependent TLR signaling, may influence the host response to *S. pneumoniae* in mice recovering from influenza in different manners. Our results in *st2^−/−^* mice demonstrate that ST2 exercises anti-inflammatory effects during both primary influenza infection and secondary *S. pneumoniae* pneumonia following influenza infection, as reflected by elevated lung levels of some cytokines. However, considering the amount of comparisons made while evaluating these cytokine levels the possibility of these differences being errors of the first kind should be taken into account, even though they seem to be limited to one time point (14 days after infection with influenza A). *St2* ^−/−^ mice displayed elevated MPO concentrations in whole lung homogenates 14 days after influenza infection (*i.e*. at the time of infection with *S. pneumoniae*) implying an enhanced activity of neutrophils at this time point since the numbers of Ly-6 positive cells in lung slides were similar in both groups. The biological significance of this small initial difference is probably limited and soon after induction of secondary bacterial infection much higher MPO concentrations were measured in the lungs. Considering that a swift induction of an airway inflammatory response contributes to effective clearance of *S. pneumoniae* from the respiratory tract [46], [47], one might have expected an improved defense in *st2* ^−/−^ mice. In contrast, ST2 deficiency was associated with a modestly enhanced growth of pneumococci in lungs of mice previously exposed to influenza, a trend that became apparent 24 hours after bacterial infection and statistically significant 48 hours after instillation with *S. pneumoniae*. Possibly, the increased lung levels of IFN-γ may have contributed to this relatively impaired antibacterial defense in *st2* ^−/−^ mice, considering that IFN-γ has been shown to inhibit pneumococcal clearance from the lungs of influenza infected mice [48]. In accordance with our present finding, stimulation of ST2 by its ligand IL-33 was previously reported to attenuate IFN-γ production by anti-CD3 stimulated T_H_1 cells in vitro [45] and murine *Trichuris muris* [49] and autoimmune encephalomyelitis in vivo [50]. We here showed that during primary influenza infection and particularly during secondary pneumococcal pneumonia following influenza infection, lung levels of IL-33 gradually increased in both WT and *st2^−/−^* mice. Possibly, this endogenous IL-33 inhibits IFN-γ production in WT mice while this effect is not present in *st2^−/−^* mice due to absence of the IL-33 receptor.

TLR7 dependent MyD88 activation has been reported to accelerate clearance of influenza in mice [12], [13], [15]. Our data indicate that the inhibitory effect of ST2 on MyD88 dependent TLR signaling does not influence viral clearance during primary influenza infection. Remarkably, *st2* ^−/−^ mice did show an accelerated clearance of residual influenza virus after instillation of *S. pneumoniae*, suggesting that the negative regulation of TLR activation mediated by ST2 only impacts the antiviral response in the presence of more abundant TLR triggering by concurrent bacterial infection. In accordance, stimulation of TLRs in the airways of mice prior to or directly after infection with a lethal dose of influenza was reported to protect against lethality [51]–[53] in conjunction with reduced viral titers [53].

Recent investigations have identified ST2 positive lineage negative lymphoid cells designated type 2 innate lymphoid cells (ILC2) in mouse and human lungs [39]–[41]. Mouse ILC2 were reported to accumulate in lungs after infection with influenza and either depletion of ILC2 or blocking ST2 resulted in loss of airway epithelial integrity and impaired airway remodeling [40]. In addition, amphiregulin, a lung ILC2 product, administered to the diseased lung was able to restore these ST2 dependent defects. We did not detect such a role for ST2 during influenza in the present investigation and amphiregulin levels did not differ between mouse strains. Notably, the earlier study used C57BL/6 mice [40], whereas we used BALB/c mice.

The reduced TLR responsiveness of immune cells harvested from influenza infected hosts [23], [24], mimics a similar phenomenon found in patients and animals with sepsis [54], [55]. In accordance with an impaired antibacterial airway defense in mice previously instilled with influenza virus, mice with sublethal polymicrobial abdominal sepsis are more vulnerable to secondary bacterial pneumonia [56], [57]. Our laboratory recently demonstrated that sepsis induced vulnerability to *Pseudomonas aeruginosa* lung infection is thwarted in the absence of ST2, as reflected by a strongly reduced bacterial growth in *st2* ^−/−^ mice [58]. Although the bacterial pathogens used in the post-sepsis [56], [57] and the current model of secondary pneumonia differed, these data suggest that ST2 plays differential roles in the pulmonary immune suppression following sepsis and influenza.

Like ST2, IL-1 receptor-associated kinase-M (IRAK-M) is an inhibitor of MyD88-dependent TLR signaling [59]. Unlike the limited role of ST2 described here, *irak-m^−/−^* mice showed a strongly enhanced detrimental inflammatory response after infection with influenza [60]. In addition, our laboratory recently reported an accelerated innate immune response in *irak-m* ^−/−^ mice upon infection with *S. pneumoniae* or *Klebsiella pneumoniae* resulting in a diminished bacterial growth [35], [61]. In contrast, we did not observe an effect of ST2 deficiency on bacterial burdens during primary *S. pneumoniae* pneumonia (data not shown) and found only a limited effect of ST2 deficiency on bacterial loads during postinfluenza pneumococcal pneumonia. Hence, these data clearly identify differential roles of two distinct negative TLR regulators in airway infection by two common respiratory pathogens.
